# Synergistic antibacterial effect of copper and silver nanoparticles and their mechanism of action

**DOI:** 10.1038/s41598-023-36460-2

**Published:** 2023-06-06

**Authors:** Grigory Vasiliev, Anna-Liisa Kubo, Heiki Vija, Anne Kahru, Denys Bondar, Yevgen Karpichev, Olesja Bondarenko

**Affiliations:** 1 Laboratory of Environmental Toxicology, National Institute of Chemical Physics and Biophysics, Akadeemia tee 23, 12618 Tallinn, Estonia; 2 Nanordica Medical OÜ, Vana-Lõuna tn 39a-7, 10134 Tallinn, Harjumaa Estonia; 3 Department of Chemistry and Biotechnology, Tallinn University of Technology, Akadeemia tee 15, 12618 Tallinn, Estonia; 4 Estonian Academy of Sciences, Kohtu 6, 10130 Tallinn, Estonia

**Keywords:** Antimicrobials, Nanotoxicology

## Abstract

Bacterial infections are one of the leading causes of death worldwide. In the case of topical bacterial infections such as wound infections, silver (Ag) has historically been one of the most widely used antibacterials. However, scientific publications have demonstrated the adverse effects of silver on human cells, ecotoxicity and insufficient antibacterial effect for the complete elimination of bacterial infections. The use of Ag in the form of nanoparticles (NPs, 1–100 nm) allows to control the release of antibacterial Ag ions but is still not sufficient to eliminate infection and avoid cytotoxicity. In this study, we tested the potency of differently functionalized copper oxide (CuO) NPs to enhance the antibacterial properties of Ag NPs. The antibacterial effect of the mixture of CuO NPs (CuO, CuO–NH_2_ and CuO–COOH NPs) with Ag NPs (uncoated and coated) was studied. CuO and Ag NP combinations were more efficient than Cu or Ag (NPs) alone against a wide range of bacteria, including antibiotic-resistant strains such as gram-negative *Escherichia coli* and *Pseudomonas aeruginosa* as well as gram-positive *Staphylococcus aureus*, *Enterococcus faecalis* and *Streptococcus dysgalactiae*. We showed that positively charged CuO NPs enhanced the antibacterial effect of Ag NPs up to 6 times. Notably, compared to the synergy of CuO and Ag NPs, the synergy of respective metal ions was low, suggesting that NP surface is required for the enhanced antibacterial effect. We also studied the mechanisms of synergy and showed that the production of Cu^+^ ions, faster dissolution of Ag^+^ from Ag NPs and lower binding of Ag^+^ by proteins of the incubation media in the presence of Cu^2+^ were the main mechanisms of the synergy. In summary, CuO and Ag NP combinations allowed increasing the antibacterial effect up to 6 times. Thus, using CuO and Ag NP combinations enables to retain excellent antibacterial effects due to Ag and synergy and enhances beneficial effects, since Cu is a vital microelement for human cells. Thus, we suggest using combinations of Ag and CuO NPs in antibacterial materials, such as wound care products, to increase the antibacterial effect of Ag, improve safety and prevent and cure topical bacterial infections.

## Introduction

The development of new antibacterials is one of the top priorities flagged by the World Health Organization and the scientific community^1^. A recent meta-analysis showed that there were 4.95 million deaths associated with antibiotic resistance in 2019 and highlighted the need for urgent action to address antibiotic-resistant bacterial infections^2^. Wound infection is one type of chronic infection that may lead to severe consequences such as limb amputation and, when unmanaged, to death. In 15–27% of cases, bacterial wound infection leads to gangrene that requires amputation of the limb^3^ demonstrating that current wound infection management strategies are inefficient and improved approaches are needed.

In the field of traditional antibiotics, the combinatory synergistic concept is currently considered as one of the most promising approaches to treat bacterial infections and avoid resistance^4^. The combination of diverse drugs allows to reduce the dose of the drugs and thus, causes fewer side effects compared to monotherapy^5^.

However, antibiotics are administered systemically and their combinations pose unpredictable pharmacokinetics profiles. Topically applied NP combinations would bypass these drawbacks of systemic antibiotics. Thus, we consider the application of synergistic NPs very promising. We hypothesize that due to the different modes of action on bacteria, Cu and Ag NPs have higher antibacterial efficacy when applied together, whereas Cu enhances the antibacterial efficacy of Ag NPs—the best metal-based antibacterials known so far. In addition, Cu is a microelement and is beneficial for wound healing, stimulating the migration of fibroblasts, promoting collagen synthesis, being essential for angiogenesis, supporting wound healing^6^ and bone regeneration^7^. Thus, Cu and Ag NP combinations could have a superior antibacterial effect, improve wound healing and therefore, be a highly beneficial treatment for infected wounds.

Separately, the antibacterial effects of both CuO and Ag NPs are well-studied. Using the keywords “silver nanoparticle* bacteria*” or “copper nanoparticle* bacteria*”, the search in PubMed® performed on 23th March 2023 retrieved 6,558 and 1,113 responses, respectively.

Antibacterial mechanisms of Ag NPs per se are relatively well understood. When in contact with bacteria, Ag NPs localize on bacterial cell wall and oxidize, leading to the concentrated Ag release at the NP-cell interface^8^. We previously showed that damage to the cell wall of gram-negative bacteria by Ag NPs occurs mainly at the plasma membrane, whereas the outer membrane of the bacterial cell was not a target of NPs^9^.

The molecular mechanisms of CuO NPs are less studied. In 2008, Heinlaan et al. showed that the toxicity of CuO NPs (EC_50_ = 79 mg/l) to bacteria *Vibrio fischeri* but also to crustaceans was due to solubilized Cu ions^10^. Notably, despite its excellent antibacterial effects, toxicity of CuO to human cells is lower compared to toxicity of Ag and Ag NPs. Since Cu is a vital microelement, in some cases CuO NPs facilitate positive effects such as improved angiogenesis and wound healing^6^. Detailed antibacterial mechanisms of CuO NPs were further studied using recombinant *E. coli*^11^. In contrast to Ag NPs that did not cause reactive oxygen species (ROS) under abiotic conditions, CuO NPs, especially positively charged ones, induced significant levels of ROS. The surface charge of NPs can be easily modified through surface functionalization and plays a role in the inactivation of bacteria. Since the cell wall of bacteria is negatively charged, positively charged NPs may adhere better to the bacterial cell walls and inactivate bacteria more efficiently.

We have studied antibacterial NPs since 2008 aiming to understand the toxicity mechanisms of NPs and to increase their efficacy and safety^1,8–10,12–14^. Recent articles and our research demonstrated that the antibacterial effect of NPs can be significantly enhanced if metal-based NPs or respective metals are used in combination. However, almost all previously published work focuses on the combined effects of metal ions and not NPs^15–18^. Among various studied combinations of metals (Ag with Cu, Zn, Co, Cd and Ni), the combination of Ag and Cu had the highest synergistic antibacterial effect against both Gram-negative and Gram-positive bacteria^19^. There is a growing interest in the synergy between Cu and Ag manifested in more published articles in the last years. Most of them described the antibacterial properties of nanoalloys of Cu/Ag^20,21^ or Cu/Ag with the addition of some other metals for example tungsten^22^. Furthermore, Jang and al. showed perfect antibacterial anti-biofilm and wound healing properties of Cu/Ag/Graphene Oxide composites in infected wound mouse-model^23^. Remarkably, despite these articles describing antibacterial synergy between Cu and Ag, we did not identify any publications systematically studying the studying the synergistic effect of NP combinations or its mechanisms.

In this study we hypothesized that the synergistic antibacterial mechanism of CuO and Ag NPs is driven by their complementary mechanisms of action: Ag NPs damage bacterial cell walls, facilitating the entrance of CuO NPs into bacteria, where CuO NPs disrupt intracellular molecules. More specifically, we first described the synergistic antibacterial effect between Ag and CuO NPs with different functional groups against various bacteria including multiresistant bacteria. Secondly, we conducted a set of tests to understand the mechanisms of the antibacterial synergy of NPs: measured the outer membrane damage, induction of ROS, bioavailability of Cu and Ag ions and charge transformation of Cu ion.

## Results and discussion

### Characterization of nanoparticles

The characterization of NPs used in this study is shown in Table 1. CuO NPs (CuO) and CuO functionalized with amino groups (CuO–NH_2_) had positive zeta-potential, CuO functionalized with carboxyl groups (CuO–COOH) had negative zeta-potential. All tested Ag NPs had strong negative zeta-potential ranging from − 56.6 mV in the case of coated silver nanoparticles (cAg) to − 27.7 mV in the case of nanosilver (nAg). In the RPMI cell culture medium (RPMI CCM) the zeta-potential of NPs was negative for all the NPs ranging from − 8.9 mV (CuO–NH_2_) to − 10.8 mV (CuO), most likely due to the adsorption of the serum proteins (so called “protein corona”, a dynamic camouflage resulting from the adherence of proteins on the surface of NPs) as suggested previously by Ivask et al.^24^. Thus, we assume that protein adsorption at least partly masked the effect of NP charges in all subsequent experiments. The dissolution among Ag NPs was the highest in the case of Ag_2_O and the lowest in the case of nAg.

**Table 1:** Characterization of nanoparticles (NPs) used in the current study.

Metal-based NPs or metal salts	Primary size, nm ± standard deviation	Hydrodynamic diameter (Dh) in MQ water, nm (pdi) ± standard deviation	Dh in RPMI cell culture medium, nm (pdi) ± standard deviation	Zeta-potential in MQ water, mV ± standard deviation	Zeta-potential in RPMI cell culture medium, mV ± standard deviation	Metal content, % ± standard deviation	Dissolution after 24 h in MQ water, % ± standard deviation
CuO	15.9 ± 5.2*	237 ± 31 (0.25)*	204 ± 13 (0.45)*	27.5 ± 1.8*	− 10.8 ± 1.4*	76.8 ± 5.7*	103 ± 0.5*
CuO − NH_2_	6.9 ± 2.2*	733 ± 252 (0.24)*	936 ± 229 (0.67)*	25.8 ± 1.3*	− 8.9 ± 0.8*	46.2 ± 4.0*	99.3 ± 0.8*
CuO − COOH	9.2 ± 2.5*	1124 ± 128 (0.35)*	303 ± 84 (0.70)*	− 12.0 ± 2.2*	− 10.2 ± 0.8*	33.6 ± 3.2*	98.9 ± 0.5*
CuSO_4_	NA	NA	NA	NA	NA	37.1 ± 4.5*	102.9 ± 0.3*
cAg	12.5 ± 4**	45.88 ± 0.21 (0.261)	61.2 ± 0.47 (0.24)	− 56.6 ± 1.91	− 9.76 ± 0.84	83.0 ± 9.8	5.24 ± 0.41
nAg	85.7 ± 29.3***	109.4 ± 1.3 (0.447)	156 ± 3.15 (0.403)	− 27.7 ± 1.65	− 10.49 ± 0.93	71.8 ± 12.0	1.1 ± 0.32
Ag_2_O	23 ± 16.8	81.265 ± 9.04 (0.604)	81.8 ± 24.21 (0.533)	− 50.97 ± 4.15	− 10.2 ± 0.2	80.1 ± 11.3	39.7 ± 12.9
AgNO_3_	NA	NA	NA	NA	NA	70.2 ± 7.95	96.7 ± 6.3

cAg, Coated silver nanoparticles; nAg, Nanosilver; Ag_2_O, Silver oxide; CuO–NH_2_, CuO coated with amino groups; CuO–COOH, CuO coated with carboxyl groups, RPMI, Roswell Park Memorial Institute medium.

*Characterization of NPs has been done previously^13^.

**Characterization of NPs has been done previously^14^.

***Characterization of NPs has been done previously^8^.

### Antibacterial synergy between Cu and Ag compounds

**Figure 1:**
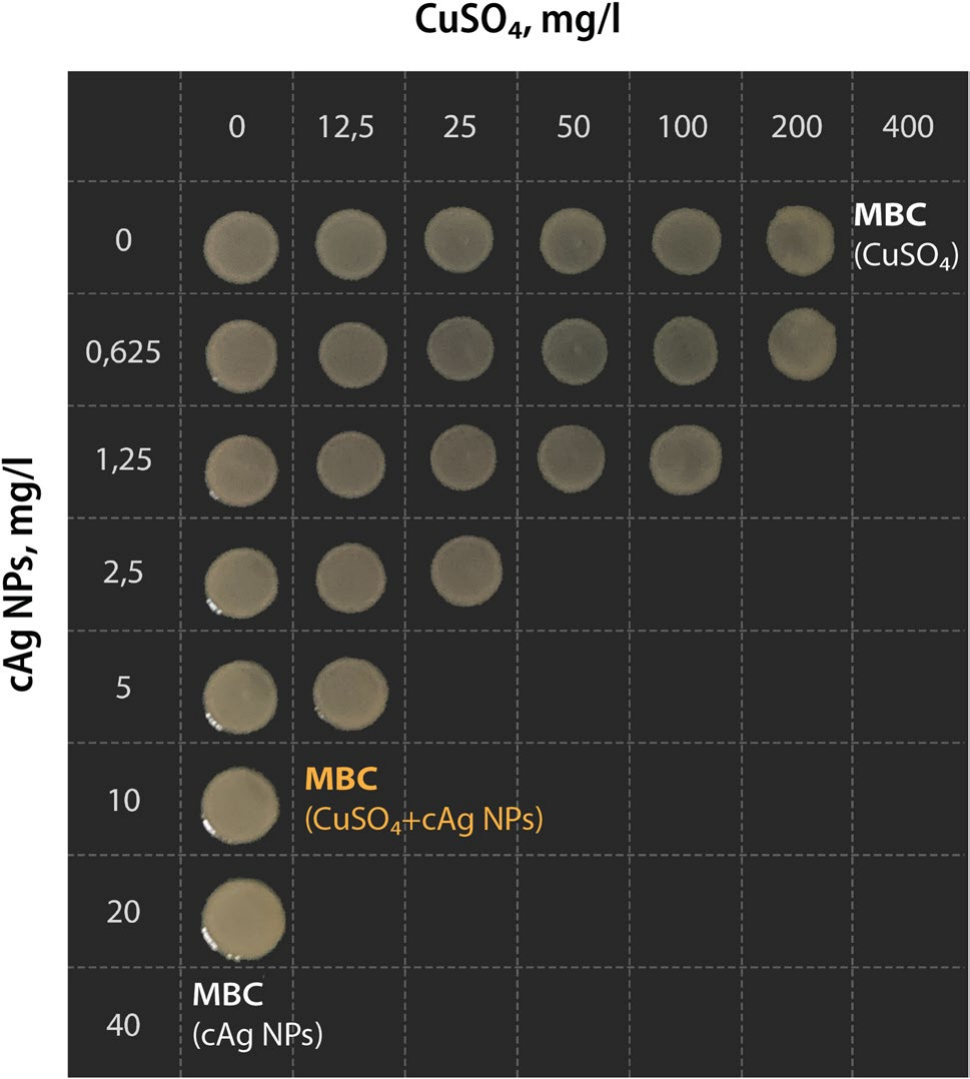
The antibacterial synergy between CuSO_4_ and cAg. *Escherichia coli* K-12 suspension was incubated with different concentrations of either Ag NPs, CuSO_4_ or their combinations in RPMI cell culture medium for 24 h. After incubation 3 μl of the bacteria-NP mixture was pipetted onto agarized broth and minimal bactericidal concentration (MBC, the lowest tested concentration yielding no visible bacterial growth after 24 h incubation at 37 ֯C in the dark) was determined. The concentrations of cAg and CuSO_4_ are shown on the axes. The minimal bactericidal concentration of CuSO_4_ and cAg were 400 mg/L and 40 mg/L respectively.

The synergistic effect was first characterized using RPMI cell culture testing medium because it contains blood serum with growth factors, which makes it similar to transudate that appears during tissue infection. An example of the synergistic antibacterial effect of NPs is shown in Fig. 1. To demonstrate the synergistic effect, we used minimum bactericidal concentration (MBC, the lowest tested concentration yielding no visible bacterial growth on agarised growth medium) as a proxy. While 40 mg/l Ag NPs or 400 mg/l CuO NPs were required to irreversibly inactivate *E. coli*, only 5 mg/l Ag NPs + 25 mg/l CuO NPs were needed when used in combination (Fig. 1).

To quantify and compare the synergistic effect between different NPs and among various bacterial strains, we introduced the term “coefficient of antibacterial synergy”, K(AbS), showing the antibacterial efficiency of NPs combinations (mix) compared to the sum of the MBC values of individual NPs and calculated as follows (Eq. 1):

1 $$K\left( {AbS} \right) = 1/\left( {\frac{{{\text{MBC}}\;{\text{of}}\;{\text{antibacteria}}\;{\text{A}}\;{\text{in}}\;{\text{mix}}}}{{{\text{MBC}}\;{\text{of}}\;{\text{antibacteria}}\;{\text{A}}\;{\text{alone}}}} + \frac{{{\text{MBC}}\;{\text{of}}\;{\text{antibacteria}}\;{\text{B}}\;{\text{in}}\;{\text{mix}}}}{{{\text{MBC}}\;{\text{of}}\;{\text{antibacteria}}\;{\text{B}}\;{\text{alone}}}}} \right)$$

Equation (1) shows the calculation of coefficient of antibacterial synergy (K(AbS) from minimal bactericidal concentrations (MBC).

Similar synergy calculation has been reported previously for metal mixtures by Vaidya et al.^16^. K(AbS) > 1 marks synergy, K(AbS) = 1 marks additive effect or K(AbS) < 1 marks antagonism.

Figure 1 demonstrates that the MBC of the mixture of cAg and CuO NPs is several times lower than the MBC of the components separately. According to formula of ${\text{K}}\left( {{\text{AbS}}} \right) = 1/\left( {\frac{{50\;{\text{mg}}/{\text{L}}}}{{400\;{\text{mg}}/{\text{L}}}} + \frac{{2.5\;{\text{mg}}/{\text{L}}}}{{40\;{\text{mg}}/{\text{L}}}}} \right)$ = 1/(1/8 + 1/16) = 5.33. Thus, in this example, the antibacterial effect of the mixture was 5.33 times higher than the sum of the antibacterial effects of the components separately.

Calculations for the mixture of 25 mg/L of CuSO_4_ + 5 mg/L of cAg result in the same K(AbS). However, K(AbS) is lower in other variations of CuSO_4_/cAg ratios (for example 200 mg/L of CuSO_4_ + 1.5 mg/L of cAg mixture). The difference between K(AbS) depending on Cu/Ag ratio is shown in Supplementary Fig. 1. The highest K(AbS) was mostly observed using the ratio from 1:1 to 12.5:1 Cu/Ag.

### Antibacterial synergy depended on the Cu component

MBC values of different Cu components alone and in the mixture with cAg in different bacteria are shown in Supplementary Table 1. MBC for different bacteria were similar, excluding *P. aeruginosa*, which was resistant to Cu compounds, despite cAg MBC for *P. aeruginosa* being similar to that of other bacteria.

**Figure 2:**
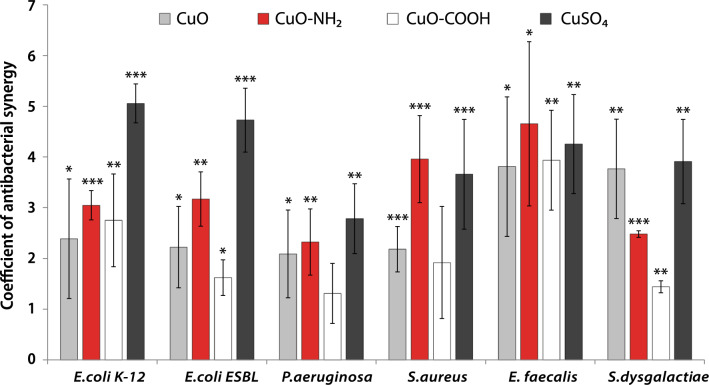
Coefficient of antibacterial synergy between cAg and copper components in different bacteria in the RPMI cell culture medium. The mean values with standard deviation are shown. *P < 0.05; **P < 0.01; ***P < 0.001. cAg, Coated silver nanoparticles; CuO Copper oxide; CuO–NH_2_, Copper oxide coated with amino groups; CuO–COOH, Copper oxide coated with carboxy groups; CuSO_4_, Copper sulphate.

Calculated K(AbS) for different bacteria is shown in Fig. 2. The evident antibacterial synergistic effect between most of the Cu compounds and Ag NPs was found (Fig. 2, Supplementary Table 1).

Among CuO NPs, the highest K(AbS) with cAg was observed with unfunctionalized CuO and especially CuO–NH_2_, both positively charged. The lowest K(AbS) was observed with negatively charged CuO–COOH, being mostly lower than 2. This suggests that the pristine charge of CuO NPs (reflected in zeta-potential in MQ water) affected antibacterial synergy. Interestingly, the charge of NPs was uniform in cell culture medium due to protein corona (Table 1). This suggests that some pristine surfaces of NPs remained available even after formation of the protein corona in cell culture medium.

The highest K(AbS) was observed for *E. faecalis* and *E. coli* (both K-12 and ESBL). It is interesting to note that *E. coli* ESBL is more resistant to antibiotics compared to *E. coli* K12 and was also more resistant to cAg in this study. However, K(AbS) values were similar in the case of these bacteria. For *E. faecalis*, high K(AbS) was observed for all combinations that may be the result of faster rate of DNA destruction by Cu components compared to Enterobacteria (such as *E. coli*)^25^.

*P. aeruginosa* had the lowest K(AbS) from all the bacteria tested. The reason for that could be the powerful antidrug efflux system and decreased outer membrane permeability^26^.

### Antibacterial synergy depended on the Ag component

We conducted additional studies with various Ag NPs and AgNO_3_ to determine which of them would have stronger antibacterial synergy with the Cu components.

**Figure 3:**
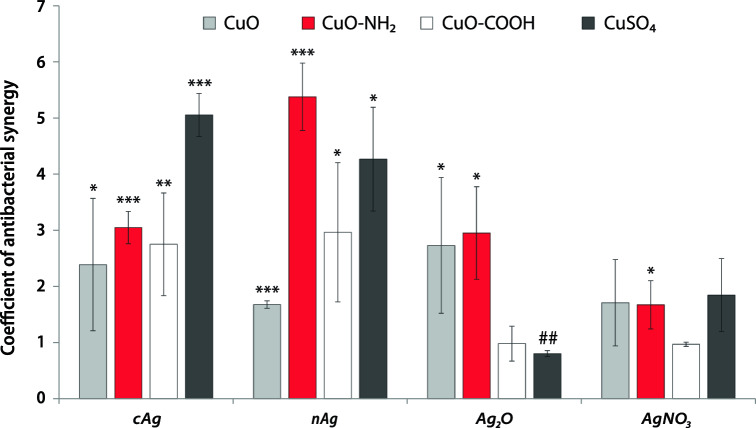
Coefficient of antibacterial synergy (K(AbS)) between different Ag and Cu components in RPMI cell culture media with *Escherichia coli* K-12 strain. *P < 0.05; **P < 0.01; ***P < 0.001, ##*P* < 0.01 antagonism. *Note* K(AbS) has been calculated as mean K(AbS) from different experiments, not from mean MBC of components in the mix or alone. The mean values with standard deviation are shown. cAg, Coated silver nanoparticles; nAg, Nanosilver; Ag_2_O, Silver oxide; AgNO_3_, Silver nitrate; CuO, Copper oxide; CuO–NH_2_, Copper oxide coated with amino groups; CuO–COOH, Copper oxide coated with carboxy groups; CuSO_4_, Copper sulphate.

Supplementary Table 2 shows the findings of MBC in *E. coli* K-12 with various Ag and Cu components in the mixture and alone. The calculated K(AbS) are shown in Fig. 3.

When mixed with Cu components, most of Ag NPs showed high K(AbS). In contrast, AgNO_3_ did not demonstrate strong antibacterial synergy with Cu components (Fig. 3, Supplementary Table 2). The highest K(AbS) were observed for three mixtures: cAg with CuSO_4_ and nAg with CuSO_4_/CuO–NH_2_. The fact that CuSO_4_ and CuO–NH_2_ were well dissolved (Table 1) and thus, were a source of Cu ions, suggests that Cu ions are required for synergy. The atypical K(AbS) result was in the mix of Ag_2_O and CuSO_4_ (no synergy). In contrast to cAg and nAg, Ag_2_O NPs were more oxidized (dissolved). These data strongly indicate that non-oxidized Ag NPs and Cu ions are both required to achieve the strong antibacterial synergistic effect.

### Antibacterial synergy depended on the bacterial medium

**Figure 4:**
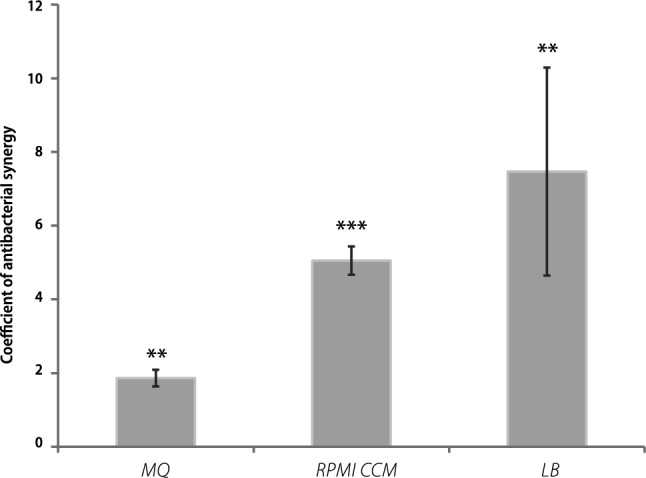
Coefficient of antibacterial synergy in different media. Coefficient of antibacterial synergy in the mixture of coated silver nanoparticles and copper sulphate in different growth media against *Escherichia coli* K-12 strain. The mean values with standard deviations are shown. *MQ* MilliQ water, RPMI.

Next, we studied whether the cultivation medium had an effect on antibacterial synergy. MBC of cAg, CuSO_4_ and their mixtures in different media is shown in Supplementary Table 3, and K(AbS) in Fig. 4. In bacterial growth medium with high content of proteins and nutrients, K(AbS) was higher. The highest K(AbS) was in LB medium and the lowest was in MQ (Fig. 4). Importantly, the growth of bacteria was the fastest in LB too (Supplementary Fig. 2). This suggests that synergy is more prominent for cells in the active and dividing phase. Most probably, in the condition of active bacterial growth, the NPs and metal ions released from the NPs have better access to intracellular space for destroying the DNA^27^ and damaging proteins. Interestingly, also traditional antibiotics inactivate bacteria less effectively in the non-growing phase, especially *S. aureus*^28^.

## The mechanisms of synergy between CuO and Ag NPs

Several additional tests were performed to understand the mechanisms of antibacterial synergy between Cu and Ag. It was hypothesized that antibacterial synergy is a function of the different modes of action of Cu and Ag. The interaction of beta-lactam antibiotics, which damage the bacterium cell membrane, and aminoglycosides, which inhibit protein synthesis in bacteria, is a well-known example of the classic synergy of antibiotics. The synergy between aminoglycosides and β-lactams has been attributed to β-lactam-mediated membrane damage leading to increased uptake of aminoglycosides^29^.

### The synergy of Cu and Ag components did not depend on bacterial outer membrane damage

**Figure 5:**
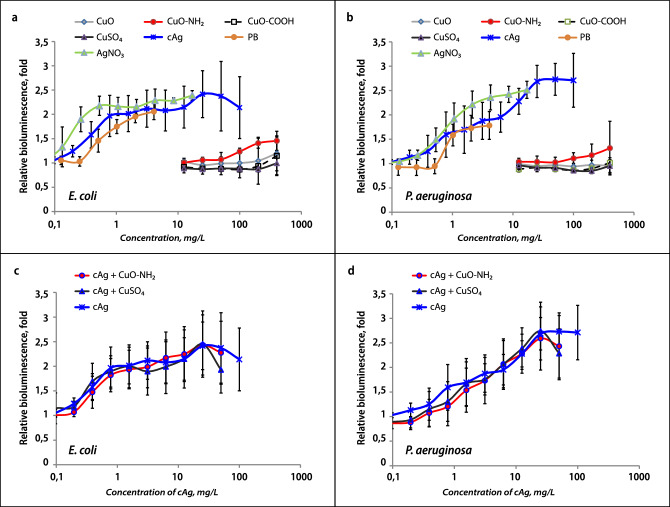
Damage of outer bacterial membrane by Ag and Cu compounds or their combination. Increase in fluorescence is indicating the outer membrane damage of *Escherichia coli* (**a**, **c**) and *Pseudomonas aeruginosa* (**b**, **d**) after 30 min of incubation with the components alone (**a**, **b**) and in the mixtures with cAg (**c**, **d**). The mean values with standard deviation are shown. cAg, Coated silver nanoparticles; AgNO_3_, Silver nitrate; CuO, Copper oxide; CuO–NH_2_, Copper oxide coated with amino groups; CuO–COOH, Copper oxide coated with carboxy groups; CuSO_4_, Copper sulphate; PB, Polymyxin B.

One hypothesis was that one of the components (Cu or Ag NPs) damages the membrane to a larger extent, allowing another component to penetrate into the cell more easily and harm the internal structures. Polymyxin B, which disrupts the outer bacterial membranes of Gram-negative cells^30^, was used as a positive control in this experiment. To verify the hypothesis, the permeability of the outer membrane of two Gram-negative bacteria, *E. coli* K-12 and *Pseudomonas aeruginosa* PAO1, was measured. The results showed that AgNO_3_ and cAg damaged the outer membrane of both bacteria (Figs. 5a,b). cAg required more time to damage the membrane compared to AgNO_3_, and *P.aeruginosa*’s membrane required more time to be damaged compared to *E. coli.* CuO NPs and CuSO_4_ did not cause remarkable membrane damage, especially in *P. aeruginosa*. This data suggests that one of the ways to inactivate cells in the case of Ag compounds is by destroying the outer membranes of the bacteria, whereas Cu compounds act on other cell structures (even high concentrations of Cu that lead to cell death in 24 h do not lead to rapid outer membrane destruction). Membrane destruction by Ag could help Cu to reach these internal cell structures in the case of Ag and Cu mixture. Relying on this data we decided to test this hypothesis by mixing Cu and Ag compounds. A test with a mixture of Cu compounds and cAg was also conducted. cAg damaging action on the membrane was unaffected by the addition of CuSO_4_ or Cu–NH_2_ (Fig. 5c,d). Tests with the addition of CuO–COOH or CuO to cAg were conducted too, and the additional destructive effect on the membrane was not detected (not shown) as well as with addition of CuSO_4_ or Cu–NH_2_. Also, adding of Cu components to Polymyxin B, which rapidly damages the outer membrane, did not enhance the effect of Polymyxin B on the membrane (data not shown). Moreover, the antibacterial synergistic effect between CuSO_4_ and Polymyxin B in *E. coli* K-12 in MBC test has not been detected (K(AbS) = 1.054 ± 0.243, data not shown). Thus, we concluded that Ag and Cu act differently on the bacterial outer membrane but their action on the outer membrane is not the main cause of antibacterial synergy.

### The synergy of Cu and Ag components did not depend on reactive oxygen species

The possibility of Cu ions to redox-cycle between Cu^+^/Cu^++^ is known as a Fenton-like reaction, and it can generate reactive oxygen species (ROS) leading to lipid peroxidation, protein oxidation and DNA damage^31^. We next hypothesized that enhanced production of ROS might be the reason of the synergy between Cu and Ag components. Indeed, CuO NPs^13^ and Ag NPs^32^ were previously shown to induce ROS. We also previously demonstrated that CuO–NH_2_ induces more ROS compared to unfunctionalized CuO and CuO–COOH^13^.

**Figure 6:**
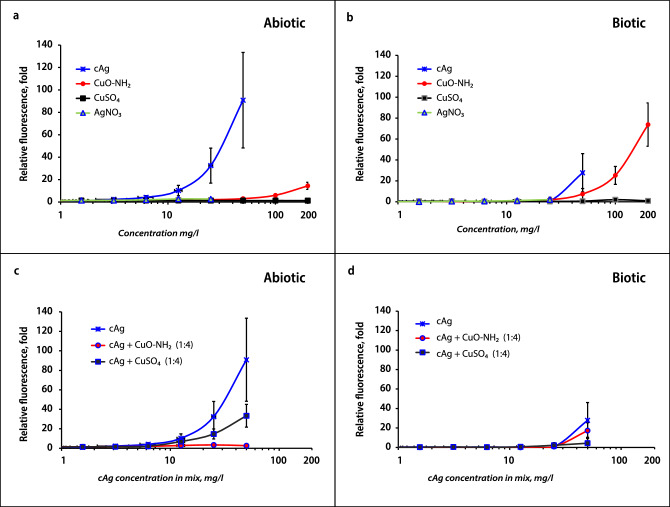
Production of reactive oxygen species (ROS) in suspensions. Measurement of induction of ROS in abiotic (**a**, **c**) and biotic (**b**, **d**) conditions. Measurement of ROS induction was performed after incubation with components alone (**a**, **b**) or in the mix of cAg and Cu components in a ratio 1:4 (**c**, **d**). cAg, Coated silver nanoparticles; AgNO_3_, Silver nitrate; CuO, Copper oxide; CuO–NH_2_, Copper oxide coated with amino groups; CuSO_4_, Copper sulphate; RFU, Relative fluorescence units.

ROS generation in this study was determined in both biotic (in presence of bacteria) and abiotic (without bacteria) conditions (Fig. 6). The highest levels of ROS were detected in individual cAg and CuO–NH_2_ suspensions (Fig. 6a,b). cAg induced the highest ROS levels in abiotic conditions (Fig. 6a), while CuO–NH_2_ in biotic conditions (Fig. 6b). No ROS was detected in the case of CuSO_4_ and AgNO_3_.

ROS in the mixture of cAg and Cu components (in a 1:4 ratio) were tested to control the hypothesis that the cAg will produce more ROS after the addition of the CuSO_4_ or CuO–NH_2_. Results showed that the addition of CuO–NH_2_ or CuSO_4_ to cAg did not enhance ROS production in both biotic and abiotic conditions (Fig. 6c,d). Moreover, antagonism has been detected (e.g., lower ROS in cAg + Cu components mixture compared to cAg alone), indicating that ROS was not the cause of antibacterial synergy.

### Silver and copper in a mixture synergically induced metal efflux genes

Since the toxicity of metal-based NPs is mostly caused by their ions^10,33^, we decided to measure intracellular Cu and Ag ions. For that, we used bioluminescent biosensor *E. coli* MC1061 pSLcueR/PDNPcopAlux, which produces luciferase in response to intracellular Cu and Ag^34^. In this bacterium, intracellular Ag and Cu ions induce the luminescence of bacteria in a dose-dependent manner.

Since the biosensor bacteria are not selective and detect both Ag and Cu ions, it was not possible to determine intracellular Ag and Cu individually. Therefore, we used the relative values and the coefficient of inductive synergy K(InS). K(InS) was calculated in the same way as the antibacterial synergy coefficient. K(InS) is the reciprocal of the sum of the ratios of bioluminescence (LC) peak concentrations of components in the mixture to LC peak concentrations alone (Eq. 2).

2 $$K\left( {InS} \right) = 1/\left( {\frac{LC\;peak\;conc\;of\;antibacterial\;A\;in\;mix}{{LC\;peak\;conc\;of\;antibacterial\;A\;alone}} + \frac{LC\;peak\;conc\;of\;antibacterial\;B\;in\;mix}{{LC\;peak\;conc\;of\;antibacterial\;B\;alone}}} \right)$$

Equation (2) shows the calculation of coefficient of inductive synergy (K(InS)) from bioluminescence (LC) peak concentrations (conc).

**Figure 7:**
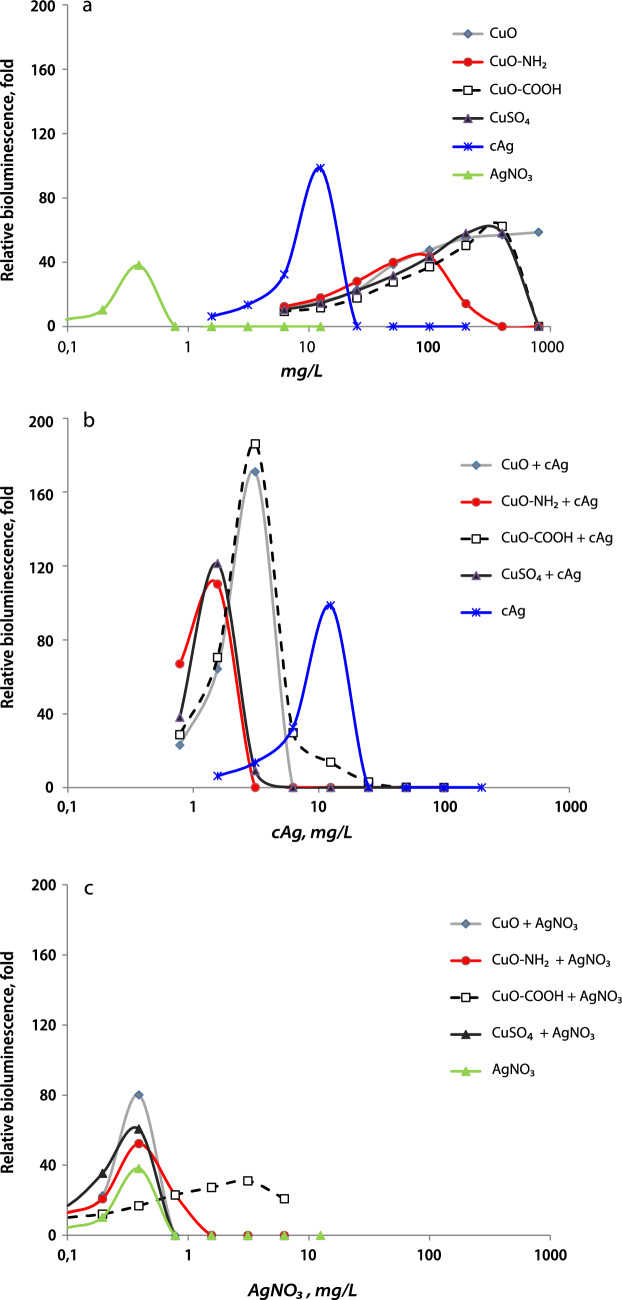
Luminescence of biosensor bacteria as reaction to components. Luminescence (LC) of *Escherichia coli* MC1061 pSLcueR/PDNPcopAlux as a reaction to 4 h incubation with components alone (**a**). The significant shift of peak LC to the left in the mix of copper components with cAg compared to cAg alone (**b**). No shift of peak LC in the mixture of copper components with AgNO_3_ compared to AgNO_3_ alone (**c**). Representative Figures are from 3 independent experiments. cAg, Coated silver nanoparticles; AgNO_3_, Silver nitrate; CuO, Copper oxide; CuO–NH_2_, Copper oxide coated with amino groups; CuO–COOH, Copper oxide coated with carboxy groups; CuSO_4_, Copper sulphate.

At first, the bioluminescence of bacteria with individual Ag and Cu components was measured (Fig. 7a). Afterwards, we mixed Cu components with cAg, and we found a significant shift in the peak of LC to the left (Fig. 7b), but no shift when mixing with AgNO_3_ (Fig. 7c). Peak LC concentrations of components separately and in the mixture are shown in Supplementary Table 4.

**Figure 8:**
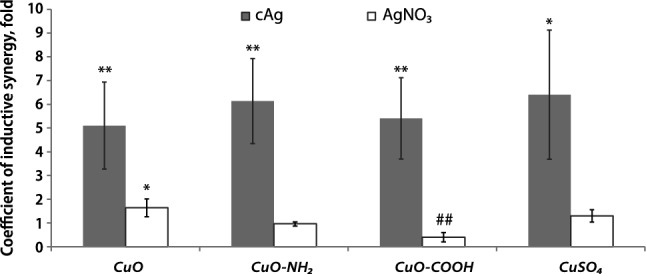
Coefficient of inductive synergy in a mixture of Cu and Ag components. Induction of bioluminescence in bacteria was higher in response to the mixture of cAg and Cu components compared to components alone. This enhanced reaction has not been detected in the mixture of AgNO_3_ and CuO NPs. *P < 0.05; **P < 0.01; ***P < 0.001, ##Antagonism *P* < 0.01. cAg, Coated silver nanoparticles; AgNO_3_, Silver nitrate; CuO, Copper oxide; CuO–NH_2_, Copper oxide coated with amino groups; CuO–COOH, Copper oxide coated with carboxy groups; CuSO_4_, Copper sulphate.

The high induction of bioluminescence in response to the intracellular concentration of Cu and Ag ions was observed in the mixtures of Cu components with cAg (Fig. 7b). Lower concentrations of cAg and Cu components in the mixtures were required for response induction. In combination of Cu components with cAg, all mixtures tested showed a high K(InS), but not in the mixtures of Cu components and AgNO_3_ (Fig. 8). The fact that K(InS) was remarkably over 1 in the case of the mixtures of cAg and Cu components revealed that biosensor bacteria sensed higher intracellular concentrations of metal ions in the mixture of cAg and Cu components compared to the sum of the components separately. This was not attributed to the membrane damage caused by cAg and better access of Cu to the inner components of the cells (Fig. 5c,d). It can be assumed that the intracellular concentration of Ag, when combined with CuSO_4_, was higher due to better dissolution of cAg in the presence of Cu^2+^. Also, the dissolution of AgNO_3_ is very high and cannot be enhanced by Cu ions, which is why we did not observe the synergistic effect between Cu components and AgNO_3_.

### Better dissolution of Ag NPs in the presence of Cu^2+^

**Figure 9:**
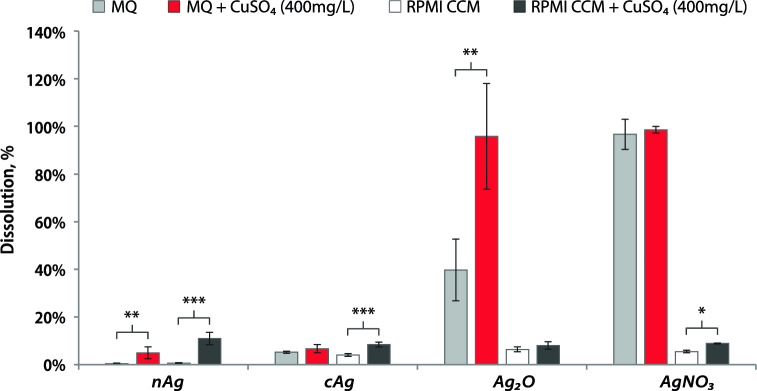
Dissolution of silver nanoparticles. Dissolution percentage of silver components (100 mg/L) in water and in RPMI CCM with and without the addition of CuSO_4_ (400 mg/L) after 24 h of incubation in a shaker at 37 °C. *P < 0.05; **P < 0.01; ***P < 0.001. cAg, Coated silver nanoparticles; nAg, Nanosilver; Ag_2_O, Silver oxide; AgNO_3_, Silver nitrate; CuSO_4_, Copper sulphate; RPMI CCM, Roswell Park Memorial Institute cell culture media.

Next, we investigated Ag NPs dissolution in water and RPMI CCM under two different conditions: with and without the addition of CuSO_4_. We found a significant difference between the dissolution of Ag NPs with and without CuSO_4_ addition (Fig. 9). In MQ water the difference of nAg and Ag_2_O dissolution was more than 4 times and 2 times respectively after CuSO_4_ addition. While in RPMI CCM the difference of nAg dissolution was more than 16 times after CuSO_4 _addition (Fig. 9). We assume that in the mix of nAg and CuSO_4_ in the water there is the effect of improving the dissolution of Ag NPs by the following Eq. 3:

3 $${\text{Cu}}_{{({\text{aq}})}}^{2 + } + {\text{Ag}}_{{({\text{s}})}} \leftrightharpoons {\text{Ag}}_{{({\text{aq}})}}^{ + } + {\text{Cu}}_{{({\text{aq}})}}^{ + }$$

Equation (3) shows the redox reaction between copper and silver components.

In RPMI, the reaction is most probably influenced by organic compounds. For example, the dissolution of AgNO_3_ in serum-containing RPMI CCM might be 100%, but most of the free Ag^+^ ions are complexed by serum proteins^35^. Because Cu ions prevent Ag ions from binding to serum proteins, there was a significant difference in the concentration of free Ag ions in a solution of AgNO_3_ in RPMI CCM with and without CuSO_4_. Both of these effects are present in a suspension of nAg and CuSO_4_ in RPMI CCM, resulting in a 16-fold increase in the amount of free Ag ions in a CuSO_4_-containing sample compared to a CuSO_4_-free sample (Fig. 9). Also, as described previously, already oxidized Ag_2_O did not have antibacterial synergy in RPMI CCM with CuSO_4_ and we did not observe better dissolution of Ag_2_O in this solvent after the addition of CuSO_4_ (Fig. 9).

### The production of antibacterial Cu^+^ ions in the mixture of Ag NPs and Cu^2+^ ions

As mentioned in the previous section (Eq. 3), Cu^+^ is produced in the mix of Ag NPs and Cu^2+^ ions. To prove the presence of Cu^+^ in the mixture, a qualitative chemical reaction was performed as described in the Methods section. In a mixture of components, the qualitative reaction proved the presence of Cu^+^, but not in nAg suspension, AgNO_3_, or CuSO_4_ solutions separately. This investigation demonstrated that the equilibrium was established as expected. Also, it has been shown previously that Cu^+^ has a higher antibacterial effect compared to Cu^2+^^36,37^, because of the ability of Cu^+^ to generate OH radicals in the presence of superoxide in a reaction analogous to the Fenton-like reaction^38^. Therefore, the formation of additional Cu^+^ ions in the solution may be among the main factors effecting the antibacterial synergy of the Ag NP/Cu^2+^ system.

### Mechanisms of antibacterial synergy of Cu and Ag nanoparticles

Commonly, synergy occurs when components have a different mechanisms of action. Although the antibacterial synergy between Ag and Cu components (mostly metals and not NPs) has been described before, the mechanisms of synergy have not been studied. Previous publications suggested some mechanisms of antibacterial synergy, whereas authors mainly hypothesized that Ag targets bacterial plasma membrane, while Cu denatures nucleic acids and other internal biomolecules and cell structures^15,16,19,22^. In contrast, in our study, we did not observe a remarkable synergy between Cu components and AgNO_3_ (Fig. 2) or Polymyxin B, which both caused remarkable membrane damage (Fig. 5).

Also, it has been proposed that Cu ions released from CuO alloys inactivate bacteria by boosting ROS production causing DNA damage and lipid peroxidation^16,19,22^. Moreover, Ag NPs anchored on CuO and Cu(OH)_2_ produced ROS in larger amounts compared to CuO^39^, but in this case, ROS production could be enhanced due to Cu(OH)_2_. We, however, did not observe significant ROS production in CuSO_4_ solution in biotic or abiotic conditions, whereas ROS production by cAg was remarkably higher. On the contrary, the addition of CuSO_4_ reduced the amount of ROS production in cAg suspension in biotic and abiotic conditions (Fig. 6).

Jang and al. (2020) showed that the release of Ag^+^ ions from NP was significantly higher in the solution with the higher concentration of Cu^2+^ ions^23^. Also, the higher release of Ag^+^ was related to the higher temperature and longer time of NP incubation^21^. This mechanism of synergy is in line with the results of our study.

Our research has shown that a combination of non-oxidized Ag NPs and Cu ions is essential for the synergistic antibacterial effect. The antibacterial synergy between Ag NPs and Cu components, according to our study, has at least three reasons. First, Cu ions facilitate the oxidation of non-oxidized Ag^0^ (Eq. 3) in a redox reaction and, as a result, better dissolution of Ag^+^ from Ag NPs in solvents in the presence of Cu^2+^ (Fig. 9). Indeed, non-oxidized Ag^0^ in NPs is needed for redox reaction with Cu^2+^ to produce Ag^+^ and Cu^+^. Better dissolution of Ag NPs leads to a higher concentration of Ag^+^ in the solvent and intracellularly in bacteria (Fig. 7), causing a stronger antibacterial effect. The second reason is the production of Cu^+^ ions in the same redox reaction (Eq. 3). Cu^+^ ions are more antibacterial compared to Cu^2+^^38^. The third reason is that free Ag ions bind to proteins in the culture medium less effectively in the presence of Cu^2+^, resulting in a larger concentration of free Ag^+^ in the solution (Fig. 9). Thus, the release of Cu^2+^ ions from CuO NPs is required for the synergistic action. All of the described processes can occur both extracellularly and intracellularly, whereas the latter is more harmful to cell homeostasis. Moreover, other factors, such as zeta-potential and NP surface functionalization might have some minor effects on the antibacterial synergy but in order to prove that, further studies are required.

## Conclusion

Ag and Cu have been used separately as antibacterial agents since ancient times. Our study showed that the antibacterial effect of the NPs mixture containing Ag and Cu components was up to six times higher than the sum of the antibacterial effects of the individual components. The mechanisms of the synergy are: better dissolution of Ag in the presence of Cu ions due to oxidation in the redox reaction, the production of more antibacterial Cu^+^ ions during the same redox reaction and less favorable binding of Ag ions to medium proteins in the presence of Cu ions. The antibacterial synergy between Ag and Cu could be an ideal solution for some medical problems where the elimination of infection is needed and where the antibacterial effect of Ag or Cu alone is not sufficient. For example, the results of the study can be used to develop combinatory nanotechnology to be used in antibacterial wound dressings to treat infected wounds. Since the treatment of some bacterial wound infections is an unsolved problem, combinatory treatment of wounds based on Cu and Ag NPs could reduce wound infection complications (such as infection-related gangrenes and amputations) and increase the quality-adjusted life years (QALY) of patients.

## Methods and materials

### Chemicals

All the chemicals were at least of analytical grade. Polymyxin B and NaCl were purchased from Sigma-Aldrich Co. (USA); AgNO_3_ from J.T. Baker (USA), CuSO_4_ from Alfa Aesar Gmbh & Co. (Germany); 2′,7′-dichlorodihydrofluorescein diacetate from Life Technologies (USA); phosphate buffered saline from Biognost (Croatia); tryptone, yeast extract and agar from LabM (UK); RPMI 1640 with glutamine and Sodium Pyruvate from Corning (USA); Fetal Bovine Serum from Gibco (USA).

### Nanoparticles (NPs)

Three types of functionalized and unfunctionalized CuO NPs were obtained from PlasmaChem GmbH (Germany). CuO NPs were synthesized by PlasmaChem by decomposition of Cu_2_CO_3_(OH)_2_, followed by the introduction of the surface groups via treatment with mercaptopropionic acid. Coated Ag NPs (cAg) were obtained from Laboratorios Argenol S. L. (Spain), uncoated Ag NPs (nAg) from Sigma-Aldrich (USA). Ag_2_O NPs (Ag_2_O) were synthesized by us as described below.

Commercial NPs were provided as dry powders, and the suspensions were freshly prepared each time before the tests at concentrations of 2000 mg compound/l in endotoxin-free Milli-Q® water (MQ). Ten milliliters of CuO NPs suspensions were vortexed and sonicated using probe sonication (Branson 450 Sonifier, USA) for 5 min with an acoustic power of 13 W corresponding to the specific energy of 3.9·105 kJ/m^3^^40^.

The morphology and primary size of all NPs except Ag_2_O were characterized in our previous studies^8,13,41^.

Ag_2_O NPs characterization by X-ray crystallography and Transmission electron microscopy was performed at the University of Tartu. Scanning transmission electron microscopy (STEM) measurements were made with Cs corrected FEI Titan Themis 200 instrument. Samples were prepared in isopropyl alcohol, sonicated and deposited onto carbon film-covered TEM grids. After evaporation of solvent, the samples were measured with bright field (BF) and high-angle annular dark-field (HAADF) detectors simultaneously.

Hydrodynamic size (Dh), polydispersity index (PDI) and zeta potential (Zeta-potential) of NPs were measured in 100 mg/l suspensions in MQ water or in Roswell Park Memorial Institute (RPMI) cell culture medium with 10% FBS and 1% sodium pyruvate (RPMI CCM) using Malvern Zetasizer (Zetasizer Nano-ZS, Malvern Instruments, UK).

The metal content of the tested compounds was determined using atomic absorption spectroscopy (AAS) (contrAA 800, Analytik Jena Ag). Materials and nanoparticles were incubated in 1 mL of concentrated HNO_3_ (Nitric Acid TraceMetal Grade 67–69%, Seastar Chemicals) for 24 h at 65֯ C. After incubation, the suspension was vortexed and diluted 1:1000 in 1% HNO_3_. The tissue and blood serum were incubated in H_2_O_2_ (Perdrogen 30%, Sigma-Aldrich) and concentrated HNO_3_ at the following ratio 1:1:8 (tissue: H_2_O_2_: HNO_3_) and incubated for 24 h at 65֯ C. After incubation, the suspension was diluted 10 times in 1% HNO_3_ and analyzed by AAS. The measurements were done in triplicate in at least two independent experiments.

### Preparation of silver oxide nanoparticles (Ag_2_O)

Analytical reagent grade (anhydrous, with purity over 99.9%) silver nitrate (5 mmol, 0.85 g) was dissolved in 50 mL of DI water and ultrasonicated for 1 min to achieve a homogeneous solution at room temperature.

After the silver nitrate was totally dissolved, sodium hydroxide (10 mmol, 0.39 g) was swiftly added to the reaction mixture at ambient conditions and ultrasonicated for 2 h.

The final solution was cooled down. Then the obtained blackish precipitate was filtered on the Whatman filter paper (Grade 5) and quenched twice with 50 mL of MQ water. The black residue was then dried at 70C for 12 h providing yields over 60%.

## Cells

### Bacterial cells

*Escherichia coli* MG1655 K-12 (*E.coli* K-12) (obtained from the *E. coli* genetic stock center, Yale University), *Escherichia coli* ESBL* (*isolated from the urine of 72 y.o. male patient with chronic prostatitis in West-Tallinn Central Hospital), *Pseudomonas aeruginosa* PAO1 (obtained from Prof. Patrick Plesiat, Besanc, France), *Streptococcus dysgalactiae* DSM 23,147 (obtained from German Collection of Microorganisms and Cell Cultures of Leibniz Institute, Braunschweig, Germany), recombinant bioluminescent *E. coli* MC1061 (pSLcueR/pDNPcopAlux, constructed in our laboratory previously by Ivask et al.^34^), *Enterococcus faecalis* ATCC 29,212 (obtained from the West-Tallinn Central Hospital) and *Staphylococcus aureus* ATCC 25,923 (obtained from the West-Tallinn Central Hospital) were stored on agarized Luria–Bertani medium (LB, 1% tryptone, 0.5% yeast extract, 0.5% NaCl, 1.5% agar) and before the tests cultivated in 3 mL of RPMI at 37 °C with shaking at 200 rpm overnight. After overnight cultivation, 400 µl of inoculum was mixed with 20 mL of RPMI CCM and incubated for 4 h and grown to the exponential phase. After the incubation, the optical density of the bacterial suspension at 620 nm (OD_620_) was measured and the suspension at desired OD_620_ was prepared. In the case of recombinant bacteria, RPMI CCM was supplemented with 100 µg/l ampicillin and 10 µg/l tetracycline to retain the bioluminescence-encoding plasmid.

## Assays

### Evaluation of minimum bactericidal concentration (MBC)

MBC was determined by the lowest concentration that kills 99.9% of the initial bacterial population using the spot test described by Suppi et al.^42^. After overnight cultivation and 4 h incubation to exponential phase, as described before, the bacterial suspension with OD_620_ 0.07 (corresponds to 2 × 10^8^ cells/mL^43^) in MQ, RPMI CCM or LB was obtained. 100 μL of bacterial suspension was added to 100 μL components alone or their mix in medium. Bacteria were exposed in RPMI CCM, LB or MQ on 96-well microplates (BD Falcon) at 30 °C for 24 h without shaking in the dark. 3 µl of incubated suspension was pipetted onto agarized LB medium and the viability of bacterial cells (formation of colonies) was visually evaluated after 24 h of incubation. Each experiment was repeated at least three times.

### Measurement of bacterial growth in different media

Bacteria were prepared for the test as described in the paragraph “Evaluation of the minimum bactericidal concentration (MBC)”. Bacterial suspension with OD_620_ 0.03 in MQ, RPMI CCM or LB was incubated for 3 h at 37 °C in a shaker and the bacterial density at OD_620_ was measured.

### Evaluation of bacterial outer membrane (OM) integrity by 1-NPN assay

The cell wall permeabilization of *E. coli* and *P. aeruginosa* by AgNO_3_, cAg, CuO, CuO–COOH, CuO–NH_2_, CuSO_4_ and Polymixin B (PB, positive control) was assayed by the cellular uptake of N-Phenylnaphthalen-1-amine (1-NPN) essentially as described by Helander and Mattila-Sandholm^44^. Differently from a hydrophilic environment, the fluorescence of 1-NPN is significantly enhanced in a hydrophobic environment (e.g., membrane lipid bilayer), rendering it an appropriate dye to probe the outer membrane (OM) integrity of gram-negative bacteria. Briefly, 50 µL of 40 µM 1-NPN and 50 µL of the tested compound in 50 mM 3-(N-morpholino)propanesulfonic acid (MOPS) adjusted with tris(hydroxymethyl)aminomethane (TRIS) base to pH 7.2 (MOPS-TRIS buffer) were pipetted into the wells of black microplates. 100 µL of bacterial suspension in 50 mM MOPS-TRIS buffer with OD = 0.5 was dispensed into each well and the fluorescence was measured after 30 min of incubation at RT (Fluoroskan Ascent FL plate luminometer; excitation/emission filters 350/460 nm). The 1-NPN cell uptake factor was calculated and presented as a ratio between the intensity of fluorescence values of the bacterial suspension incubated with and without test compounds. At least three independent experiments were performed in technical duplicates.

### *CopA* gene induction of luminescent bacteria

Induction of luminescence in bacteria by intracellular Ag and Cu ions was performed using recombinant biosensor bacteria *Escherichia coli* MC1061 (pSLcueR/pDNPcopAlux). The response of recombinant *E. coli* to intracellular Ag and Cu ions is mediated via CueR activator protein and its regulated *copA* promoter that is fused to the bioluminescence encoding genes^34^. Therefore, in the sub-toxic region, the presence of intracellular Ag and Cu ions leads to the increase of bioluminescence of these recombinant bacteria in a dose-dependent manner.

The preparation of test bacteria and the procedure of the biosensor assay were analogous to the bacterial growth inhibition assay with one exception: the growth medium of bioluminescent Ag-biosensor *E. coli* MC1061 (pSLcueR/pDNPcopAlux) was supplemented with 100 µg/l ampicillin and 10 µg/l tetracycline during overnight cultivation to maintain the recombinant plasmids. Orion II plate luminometer (Berthold Detection Systems) was used for the bioluminescence (BL) measurement. 100 µl of bacterial suspension with OD_620_ 0.1 was exposed to components or their mix in RPMI CCM (sample) or RPMI CCM (background) at 30 °C for 4 h. Dose–response curves of the Ag and Cu biosensor were obtained by plotting the applied concentrations of Cu and Ag against the bioluminescence of Ag/Cu-biosensor (as fold induction) in respective samples. The concentration with the highest bioluminescence value from each component has been marked as the peak of LC. Fold induction was calculated as follows:

$$Induction\left( {fold} \right) = \frac{{BL_{sample} }}{{BL_{background} }}$$

where BL_sample_ is the bioluminescence of Ag/Cu-biosensor in the sample and BL_background_ is the background bioluminescence considering darkening of luminescence due to NPs.

### Quantification of reactive oxygen species (ROS)

Since presence of cells may influence generation and neutralization of ROS, ROS was quantified in both, biotic (in the presence of cells) and abiotic (without cells) conditions.

To study cellular (biotic) ROS production, fresh stock of 2′,7′-dichlorodihydrofluorescein diacetate (H_2_DCFDA) was dissolved in ethanol to a concentration of 0.6 mg/mL and subsequently diluted in 0.1 M sodium phosphate buffer. 100 µL of component in MQ water was pipetted into the wells. Then, 100 µL of bacterial culture (*E. coli* K-12 at OD_620_ = 0.1) in MQ water was added. The test plates were incubated at 30 °C for 4 h. After incubation 100 μL of the mixture was aliquoted into black non-transparent 96 well plates, and H_2_DCFDA was added to the cells at a final concentration of 1.5 µg/mL. After 30 min of incubation, the fluorescence was measured using a microplate reader (excitation/emission at 485/527 nm). In the assay, H_2_DCFDA diffuses through the cell membrane and is processed by intracellular esterases to a non-fluorescent dichlorofluorescin (DCFH). DCFH is then converted to the highly fluorescent 2′,7′-dichlorofluorescein (DCF) due to the presence of intracellular ROS.

To study ROS in abiotic conditions, the acetate group of H_2_DCFDA was cleaved by 0.01 M NaOH at RT for 30 min. The dye was diluted with 0.1 M Na-phosphate buffer to a concentration of 24 µg/mL and then added to each well in the black nontransparent 96 well plates, to yield a final dye concentration of 12 µg/mL. The results were divided by the blank and presented as relative light units (RFUs).

### Determination of dissolution of the components

For the dissolution analysis, 100 mg/l cAg, Ag_2_O, nAg, AgNO_3_, CuSO_4_ (a recovery control) were incubated in MQ water or in RPMI CCM at 37 °C for 24 h and afterwards centrifuged at 320,000 × g for 30 min (Beckman Coulter ultracentrifuge). After centrifugation, the supernatants were collected and the silver concentration analyzed by AAS (contrAA 800, Analytik Jena Ag). The percentage of NP dissolution was calculated according to the silver concentration in the supernatant after centrifugation, 100 mg/l was taken as 100%. The measurements were done in triplicate in at least two independent experiments.

### Detection of Cu^+^ in the suspension

The detection of Cu^+^ was conducted using the iodometric method^45^. The Cu^2+^ was masked by a complex formation with potassium oxalate; then, the Cu^+^ was oxidized to Cu^2+^ by potassium iodate in the presence of 2 M hydrochloric acid using the 1% starch solution as an indicator of the presence of iodine, which appears when the reaction is completed.

### Statistical analysis

*P* values using Student’s t-test, standard deviations and mean values were calculated in Microsoft Excel.

## Supplementary Information

Supplementary Information.

## Data Availability

The data used and/or analyzed during the current study is available from the corresponding author on reasonable request.
