# Supplementary material for: Synergistic antibacterial effect of copper and silver nanoparticles and their mechanism of action
Source: Sci Rep. 2023 Jun 6;13:9202. doi: 10.1038/s41598-023-36460-2 (PMC10244331; doi:10.1038/s41598-023-36460-2)
Supplement: Supplementary file 1 — Supplementary Information. [file 41598_2023_36460_MOESM1_ESM.docx]

**Supplementary information** for

SYNERGISTIC ANTIBACTERIAL EFFECT OF COPPER AND SILVER NANOPARTICLES AND THEIR MECHANISM OF ACTION

*Grigory Vasiliev^1,2,3^, Anna-Liisa Kubo^1,2^, Heiki Vija^1^, Anne Kahru^1,4^, Denys Bondar^3^, Yevgen Karpichev^3^, Olesja Bondarenko^1,2*^*

^1^ Laboratory of Environmental Toxicology, National Institute of Chemical Physics and Biophysics, Akadeemia tee 23, Tallinn, 12618, Estonia

^2^ Nanordica Medical OÜ, Vana-Lõuna tn 39a-7, Tallinn, Harjumaa 1013, Estonia

^3^ Department of Chemistry and Biotechnology, Tallinn University of Technology, Akadeemia tee 15, Tallinn, 12618 Estonia

^4^ Academy of Sciences, Kohtu 6, Tallinn 10130, Estonia

* Corresponding author

Contact: (+372) 6398382, olesja.bondarenko@kbfi.ee

Supplementary Table 1. The results of Minimal Bactericidal Concentration (MBC) of components alone (coated silver nanoparticles (cAg), copper oxide (CuO), copper oxide coated with amino groups (CuO-NH_2_), copper oxide coated with carboxy groups (CuO-COOH) and copper sulphate (CuSO_4_)) and in the most effective mixture. Note: Coefficient of antibacterial synergy (K(AbS)) has been calculated as the average K(AbS) from different experiments and do not fully correspond to the calculation based on average MBCs of components in mix or alone.

| **Bacterium (Gram staining)** | **MBC alone ± standard deviation (mg/l)** | **MBC(CuX) in mix ± standard deviation (mg/l)** | **MBC(cAg) in mix ± standard deviation (mg/l)** | **K(AbS) ± standard deviation** |
| --- | --- | --- | --- | --- |
| ***Escherichia coli* K-12 (G-)** |  |  |  |  |
| cAg | 34.04 ± 13.1 |  |  |  |
| CuO | 215.4 ± 114.4 | 41.67 ± 14.43 | 7.441 ± 2.577 | 2.387 ± 1.179 |
| CuO-NH_2_ | 157.1 ± 51.4 | 18.75 ± 7.22 | 5.208 ± 3.348 | 3.047 ± 0.289 |
| CuO-COOH | 350 ± 90.5 | 12.5 ± 0 | 11.90 ± 5.16 | 2.75 ± 0.915 |
| CuSO_4_ | 217.6 ± 72.8 | 22.5 ± 16.3 | 2.679 ± 0.998 | 5.055 ± 0.383 |
| ***Escherichia coli* ESBL (G-)** |  |  |  |  |
| cAg | 45.83 ± 9.73 |  |  |  |
| CuO | 133.3 ± 57.7 | 41.67 ± 14.43 | 7.292 ± 4.774 | 2.222 ± 0.801 |
| CuO-NH_2_ | 100 ± 0 | 20.83 ± 7.22 | 5.208 ± 1.804 | 3.17 ± 0.536 |
| CuO-COOH | 333.3 ± 115.5 | 58.33 ± 38.19 | 20.83 ± 7.22 | 1.621 ± 0.352 |
| CuSO_4_ | 200 ± 0 | 25 ± 0 | 4.167 ± 1.804 | 4.727 ± 0.63 |
| ***Pseudomanas aeruginosa* PAO1 (G-)** |  |  |  |  |
| cAg | 28.91 ± 15.63 |  |  |  |
| CuO | 1400 ± 400 | 43.75 ± 12.5 | 15.63 ± 10.83 | 2.089 ± 0.867 |
| CuO-NH_2_ | 200 ± 122.5 | 20 ± 6.85 | 8.75 ± 3.423 | 2.324 ± 0.653 |
| CuO-COOH | 800 ± 0 | 34.38 ± 18.75 | 21.88 ± 6.25 | 1.31 ± 0.592 |
| CuSO_4_ | 720 ± 178.9 | 20 ± 6.85 | 8.75 ± 3.423 | 2.783 ± 0.687 |
| ***Staphylococcus aureus* ATCC 25923 (G+)** |  |  |  |  |
| cAg | 16.25 ± 5.88 |  |  |  |
| CuO | 60 ± 22.36 | 17.5 ± 6.85 | 2.969 ± 2.096 | 2.18 ± 0.447 |
| CuO-NH_2_ | 80 ± 27.39 | 12.5 ± 7.65 | 1.875 ± 0.699 | 3.958 ± 0.858 |
| CuO-COOH | 110 ± 54.8 | 11.25 ± 2.8 | 8.125 ± 4.193 | 1.918 ± 1.106 |
| CuSO_4_ | 90 ± 22.36 | 16.25 ± 8.39 | 1.875 ± 0.699 | 3.659 ± 1.083 |
| ***Enterococcus faecalis* ATCC 29212 (G+)** |  |  |  |  |
| cAg | 58.33 ± 19.46 |  |  |  |
| CuO | 233.3 ± 152.8 | 29.17 ± 19.09 | 9.375 ± 5.413 | 3.81 ± 1.374 |
| CuO-NH_2_ | 266.7 ± 115.5 | 12.5 ± 0 | 10.42 ± 3.61 | 4.655 ± 1.62 |
| CuO-COOH | 400 ± 0 | 16.67 ± 7.22 | 12.5 ± 0 | 3.936 ± 0.983 |
| CuSO_4_ | 200 ± 0 | 20.83 ± 7.22 | 8.333 ± 3.608 | 4.254 ± 0.977 |
| **Streptococcus dysgalacticae DSM 23147 (G+)** |  |  |  |  |
| cAg | 10.07 ± 3.76 |  |  |  |
| CuO | 133.3 ± 57.7 | 16.67 ± 7.22 | 1.302 ± 0.451 | 3.766 ± 0.979 |
| CuO-NH_2_ | 133.3 ± 57.7 | 15.625 ± 13.26 | 3.125 ± 0 | 2.481 ± 0.065 |
| CuO-COOH | 200 ± 0 | 12.5 ± 0 | 6.25 ± 0 | 1.44 ± 0.119 |
| CuSO_4_ | 133.3 ± 57.7 | 12.5 ± 0 | 1.563 ± 0 | 3.909 ± 0.832 |

Supplementary Table 2. The results of mean ± standard deviation of Minimal Bactericidal Concentration of components alone and in the most effective mixture in RPMI cell culture media with *Escherichia coli* K-12. Abbreviations: coated silver nanoparticles (cAg), nanosilver (nAg), Silver oxide (Ag_2_O), Silver nitrate (AgNO_3_), copper oxide (CuO), copper oxide coated with amino groups (CuO-NH_2_), copper oxide coated with carboxy groups (CuO-COOH) and copper sulphate (CuSO_4_).

| Ag compound  Cu compound | **None ± standard deviation** | **cAg, mg/L ± standard deviation** | **nAg, mg/L ± standard deviation** | **Ag_2_O mg/L ± standard deviation** | **AgNO_3_, mg/L ± standard deviation** |
| --- | --- | --- | --- | --- | --- |
| none |  | 0 | 0 | 0 | 0 |
|  |  | 34.04 ± 13.1 | 1333 ± 428 | 66.67 ± 24.62 | 10.16 ± 8.20 |
| CuO, mg/L | 215.4 ± 114.4 | 41.67 ± 14.43 | 68.75 ± 37.5 | 33.33 ± 14.43 | 54.17 ± 43.9 |
|  | 0 | 7.441 ± 2.577 | 125 ± 50 | 12.5 ± 0 | 2.744 ± 3.385 |
| CuO-NH_2_, mg/L | 157.1 ± 51.4 | 18.75 ± 7.22 | 25 ± 0 | 25 ± 21.65 | 37.5 ± 42.08 |
|  | 0 | 5.208 ± 3.348 | 87.5 ± 25 | 12.5 ± 0 | 2.508 ± 3.646 |
| CuO-COOH, mg/L | 350 ± 90.5 | 12.5 ± 0 | 62.5 ± 43.3 | 200 ± 0 | 206.3 ± 274 |
|  | 0 | 11.90 ± 5.16 | 350 ± 100 | 16.67 ± 7.217 | 4.350 ± 3.174 |
| CuSO_4_, mg/L | 217.6 ± 72.8 | 22.5 ± 16.3 | 31.25 ± 12.5 | 266.7 ± 115.5 | 12.5 ± 0 |
|  | 0 | 2.679 ± 0.998 | 100 ± 0 | 12.5 ± 7.217 | 3.646 ± 3.646 |

Supplementary Table 3. Coefficient of antibacterial synergy (K(AbS)) Minimal Bactericidal Concentration (MBC) of components alone and in the most effective mixture with different Cu and Ag components in E.coli K-12. Abbreviations: MQ (MilliQ water), coated silver nanoparticles (cAg), nanosilver (nAg), Silver oxide (Ag_2_O), Silver nitrate (AgNO_3_), copper oxide (CuO), copper oxide coated with amino groups (CuO-NH_2_), copper oxide coated with carboxy groups (CuO-COOH) and copper sulphate (CuSO_4_).

| **MQ** | **none** | **MBC of cAg,mg/L** | **K(AbS)** |
| --- | --- | --- | --- |
| none | 0 | 0.146 ± 0.095 |  |
| MBC of CuSO4, mg/L | 0.167 ± 0.72 | 0.0729 ± 0.048 | 1.861 ± 0.227 |
|  |  | 0.0107 ± 0.0085 |  |
| **RPMI CCM** |  | | |
| none |  | 34.04 ± 13.1 |  |
| MBC of CuSO4, mg/L | 217.6 ± 72.8 | 22.5 ± 16.3 | 5.055 ± 0.383 |
|  |  | 2.679 ± 0.998 |  |
| **LB** |  | | |
| none |  | 83.33 ± 28.87 |  |
| MBC of CuSO4, mg/L | 1600 ± 0 | 116.7 ± 76.4 | 7.467 ± 2.822 |
|  |  | 5.208 ± 1.804 |  |

Supplementary Table 4. Toxicants concentrations of bioluminiscense peaks of Escherichia coli MC1061 pSLcueR/PDNPcopAlux after 4 hours of incubation with toxicants separately and in mix. Abbreviations: coated silver nanoparticles (cAg), silver nitrate (AgNO_3_), copper oxide (CuO), copper oxide coated with amino groups (CuO-NH_2_), copper oxide coated with carboxy groups (CuO-COOH), copper sulphate (CuSO_4_).

| Ag compound  Cu compound | **None ± standard deviation** | **cAg, mg/L ± standard deviation** | **AgNO_3_, mg/L ± standard deviation** |
| --- | --- | --- | --- |
| none |  |  |  |
|  |  | 12.25 ± 4.79 | 0.744 ± 0.351 |
| CuO, mg/L | 614.4 ± 0 | 9.6 ± 0 | 30.72 ± 10.51 |
|  |  | 3.125 ± 0 | 0.397 ± 0.136 |
| Cu-NH_2_, mg/L | 41.58 ± 10.33 | 2.888 ± 0 | 18.48 ± 6.33 |
|  |  | 1.563 ± 0 | 0.397 ± 0.136 |
| CuO-COOH, mg/L | 134.4 ± 0 | 3.864 ± 0.939 | 67.2 ± 0 |
|  |  | 2.813 ± 0.49 | 1.588 ± 0.543 |
| CuSO_4_, mg/L | 74.2 ± 0 | 3.246 ± 1.27 | 14.84 ± 5.08 |
|  |  | 2.188 ± 0.599 | 0.397 ± 0.136 |


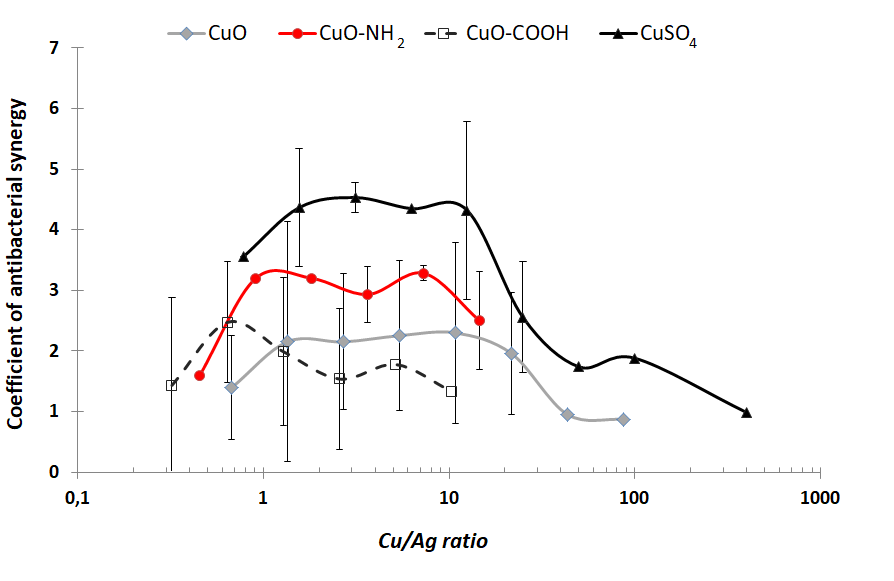


Supplementary Figure 1. Coefficient of antibacterial synergy between coated silver nanoparticles (cAg) and copper components (copper oxide (CuO), copper oxide coated with amino groups (CuO-NH_2_), copper oxide coated with carboxy groups (CuO-COOH) and copper sulphate (CuSO_4_)) depending to Cu/Ag metal ratio in mixture.

*
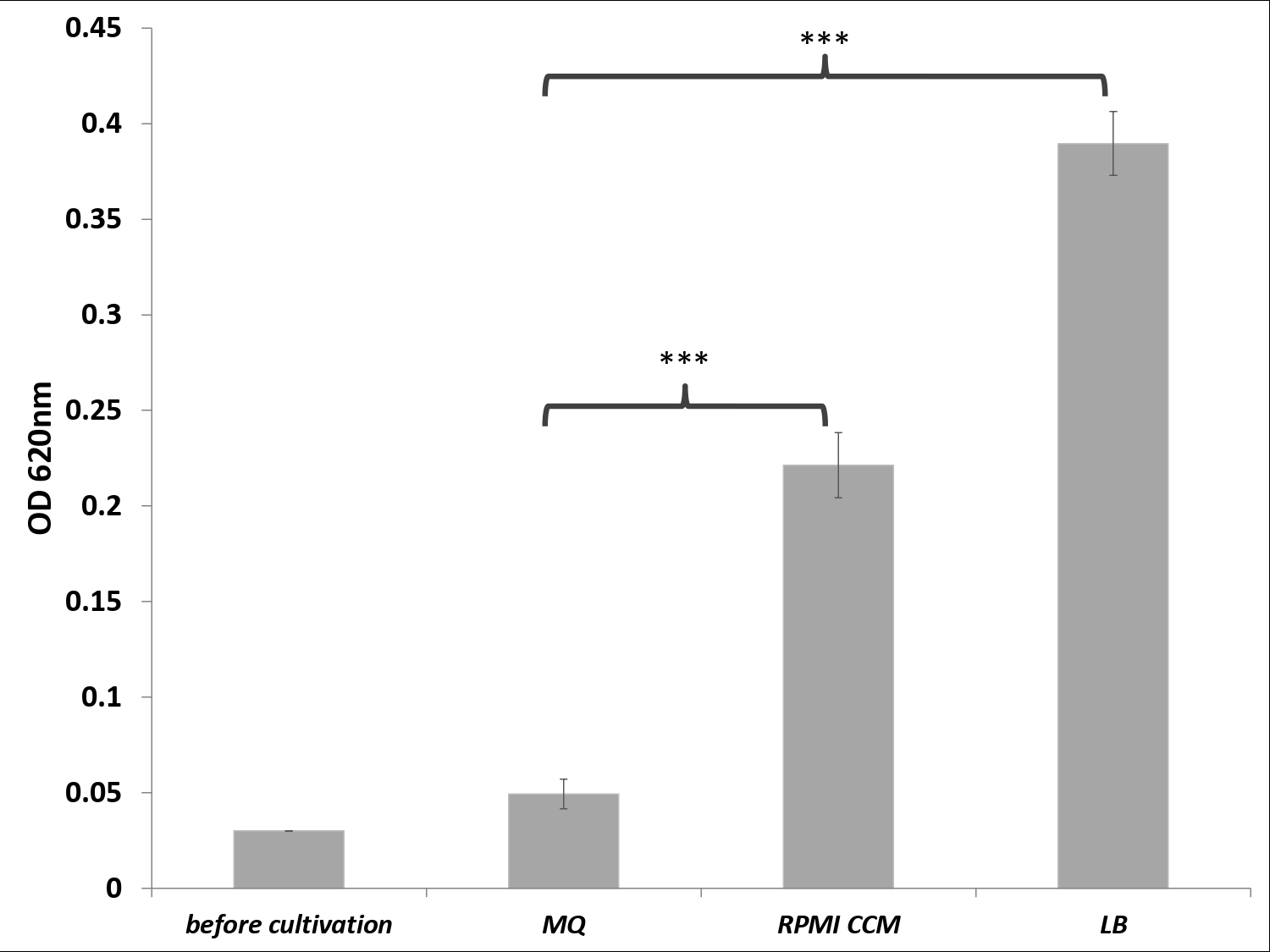
*

Supplementary Figure 2. The optical density of suspension before cultivation and after 3h in different media. The mean values with standard deviations are shown. Abbreviations: MQ - MilliQ water, RPMI CCM - Roswell Park Memorial Institute culture cell medium, LB - Luria-Bertani broth.* - P < 0,05; ** - <0,01; *** - < 0,001
